# Assessing the external validity of mobile phone surveys for monitoring ITN coverage indicators: a comparison with household surveys in Tanzania

**DOI:** 10.1186/s12936-025-05533-0

**Published:** 2025-08-28

**Authors:** Matt Worges, Ruth Ashton, Janna Wisniewski, Paul Hutchinson, Hannah Koenker, Tory Taylor, Hannah Metcalfe, Ester Elisaria, Charles Dismas Mwalimu, Peter Gitanya, Frank Chacky, Josh Yukich

**Affiliations:** 1 Celia Scott Weatherhead School of Public Health and Tropical Medicine, Tulane University, New Orleans, LA USA; 2 Tropical Health, Tel Aviv, Israel; 3 Center for Applied Malaria Research and Evaluation, Celia Scott Weatherhead School of Public Health and Tropical Medicine, Tulane University, New Orleans, LA USA; 4 Tropical Health, Baltimore, MD USA; 5 Carolina Population Center, University of North Carolina at Chapel Hill, Chapel Hill, NC USA; 6 Viamo, Dar Es Salaam, Tanzania; 7 Ifakara Health Institute, Mikocheni, Keko Avenue. Dar Es Salaam Branch, Dar Es Salaam, Tanzania; 8 National Malaria Control Programme, Ministry of Health, Dodoma, Tanzania

**Keywords:** ITN, Mobile phone survey, Random digit dial, Coverage indicators, External validity, Bias, Tanzania

## Abstract

**Background:**

Insecticide-treated net (ITN) coverage indicators allow country malaria programmes to understand the overall coverage of their ITN distribution activities and can be used to forecast the need for additional ITN procurement and to plan future distribution campaigns. As a result, more frequent data collection could better guide programme strategies, particularly for those strategies operating on an annual framework; however, the high costs and infrequency of national-scale, household surveys limit their practicality for ongoing monitoring. Due to the rapid growth trend of mobile phone ownership in low- and middle-income countries in recent years, mobile phone-based surveys (MPS) have emerged as a comparatively inexpensive alternative to large-scale household surveys.

**Methods:**

Contemporaneous to the 2017 Malaria Indicator Survey and the 2022 Demographic and Health Survey, a random digit dial and list-based MPS were conducted in Tanzania, respectively. All surveys allowed for the calculation of the same ITN coverage indicators at the regional level which permitted an assessment of the external validity of the MPS.

**Results:**

Mean measures of absolute bias, as derived from Bland–Altman analyses, for survey raked estimates of households with at least one bed net of any type and household population with access to a bed net of any type were negative for both surveys. These MPS estimates were, on average, consistently lower than corresponding estimates from the household surveys by 7–8 percentage points. Proportion of households with at least one bed net of any type for every two people was unbiased with measures of 0.02 [95% CI − 0.04, 0.08] for the initial MPS and 0.02 [95% CI − 0.03, 0.06] for the 2022 MPS, following survey raking, relative to the household surveys.

**Conclusions:**

MPS consistently underestimated household ITN access compared to contemporaneous household surveys, but the direction and magnitude of bias were stable across years. While this limits their use as a direct substitute for household surveys, MPS may still offer timely, conservative signals of declining coverage where other data are delayed or unavailable. With further validation and potential adjustment for bias, MPS could complement traditional monitoring approaches, especially in contexts where rapid data are needed to guide programme decisions.

## Background

Since 2000, the use of insecticide-treated nets (ITNs) has played an important role in reducing the global malaria burden and constitutes a core malaria prevention tool [1]. Numerous studies and field efficacy trials in sub-Saharan Africa have demonstrated that the use of ITNs significantly contributes to reducing malaria morbidity and mortality [2–7]. Research estimates suggest that the odds of ITN users contracting malaria were 56% less than non-users [8]. Since 2000, the Tanzanian government has organized the distribution of ITNs through routine distribution at health facilities and as part of the Expanded Programme on Immunization, via mass and targeted distribution campaigns, and through an additional school-based distribution channel beginning in 2013 [9–14]. Population-level indicators of ITN coverage have primarily been monitored through nationally representative household surveys. Between 2004 and 2022, the Tanzanian government conducted seven standard or interim Demographic and Health Surveys (DHS), averaging one survey every 2.7 years, each of which included a bed net sub-module to help monitor ITN ownership, access, and use indicators [15–21].

Four indicators are recommended for measuring ITN availability and use—two are at the household level (proportion of households with at least one ITN and proportion of households with at least one ITN for every two people) and two are at the population level (proportion of the population with access to an ITN in their household and proportion of the population that slept under an ITN the previous night) [22]. The proportion of households with at least one ITN demonstrates reach across the target population while the proportion of households with at least one ITN for every two people gives a sense of whether households have sufficient ITNs. The latter indicator assumes that if one ITN is given for every two people in a household then all household members have an opportunity to use an ITN, often referred to as “universal coverage” [23, 24]. The proportion of the population with access to an ITN in their household is an interpretation of depth of coverage and serves as an estimate of individuals who could have used an ITN given the assumption of two people per ITN. The fourth indicator is simply an estimate of ITN use in the population. Taken together, these indicators allow country malaria programmes to understand the overall coverage of their ITN distribution activities and as such can be used to forecast the need for additional ITN procurement as well as to plan future distribution campaigns. Consequently, higher frequency monitoring could be helpful to guide programme strategies, particularly the school net programme which uses an annual planning and procurement cycle; however, national-scale, face-to-face household surveys are expensive and occur on a relatively infrequent basis [25]. Further, household surveys may be difficult or too risky to implement in non-permissive environments and results are not immediately available due to the complexity of cleaning and analyzing such large volumes of data (generally within 12 months after the end of fieldwork for DHS surveys) [26, 27].

Due to the rapid growth trend of mobile phone ownership in low- and middle-income countries (LMIC) in recent years, mobile phone-based surveys (MPS) have emerged as a comparatively inexpensive alternative to large scale population-based household surveys [28–32]. In Tanzania, there were approximately 33 million unique subscribers to mobile network operators in 2022—an increase from 10.4 million in 2008 [33–35]. As of 2022, 83.1% of surveyed households in Tanzania reported any member of the household owning a mobile phone, though ownership is still more common among men, urban dwellers, those who have at least completed primary education, and those in higher socio-economic status households [21].

In LMIC settings, a potentially viable MPS option is the use of random digit dialing (RDD) [36–38]. RDD surveys target respondents through non-list-based random sampling using a strategy where each telephone number has an equal probability of selection [39]. Another MPS strategy uses a list-based approach where a bank of phone numbers is provided by a mobile network operator, for example.

Only one RDD survey implemented in 2017 in Ghana was identified in the published literature that specifically focused on comparing ITN use indicators to participant responses from household surveys (i.e., the 2014 Ghana DHS) [40]. Such a large difference in time points between these surveys makes it difficult to truly assess the external validity of the MPS considering seasonal changes in ITN use, ITN life span and durability, proximity of the surveys to ITN distribution events, and changes in family structure which could affect how bed nets are shared/distributed within a household. The authors of the Ghana paper did not use survey raking (i.e., a method to align sample estimates with known population distributions) which may have impacted their ability to align MPS estimates with the population's true demographic, geographic, and other characteristics.

The purpose of the current study is to describe the external validity of a 2017 and 2022 MPS conducted in Tanzania which were designed to collect information on ITN coverage indicators. MPS results adjusted through survey raking were evaluated against corresponding indicator values calculated from the contemporaneously conducted 2017 Tanzania Malaria Indicator Survey (MIS) and 2022 DHS (when referred to jointly, a default designation of ‘DHS’ is used throughout this paper). Note that in the context of comparing DHS and LMIC-based MPS, DHS results serve as the reference standard due to superior representativeness stemming from high-quality methods and, in some cases, the ability to verify the existence of ITNs in the household. The current study expands upon the limited previous research by broadening the scope of assessed ITN indicators in a different country context, more closely aligning MPS and household survey time points, and weighting results through survey raking.

## Methods

### 2017/2018 surveys

The 2017 MIS provided malaria-specific estimates for basic demographic and health indicators including Roll Back Malaria Monitoring and Evaluation Reference Group (RBM-MERG) ITN indicators. Data collection for the 2017 Tanzania MIS took place from October 2017 to December 2017. The RDD survey immediately followed running from mid-January to March 2018. The RDD survey collected data elements similar to those of the 2017 MIS in order to replicate the calculation of key ITN coverage indicators.

Using Tanzania’s 12-digit mobile number structure, the RDD process relied on a random number generator to create and place calls to potential phone numbers. Numbers dialed within Tanzania began with 255 (the country code), followed by a three-digit secondary prefix of 51 different possibilities, and the next six digits were randomly generated. Voice prompts, pre-recorded in Kiswahili, were used to guide participants through the survey. The specific MPS approach used is referred to as telephone-audio computer-assisted self-interviewing (TACASI) survey, which is a form of an interactive voice response (IVR) survey.

Individuals who received a phone call through the RDD survey first heard a standard consent script notifying them that what they were hearing was part of a malaria control research study. Consenting participants were then asked questions designed to collect information on household size, household assets, and ITN ownership/use. Respondents were not able to be geolocated based on their phone numbers so household location was collected through a series of filtering questions used to obtain region of residence. Repeat calls were placed to numbers with no initial response and for participants who prematurely broke off from the survey. Participant phone numbers were not included in the final datasets and no personally identifying information was solicited from respondents.

### 2022 surveys

The second MPS was also conducted using a TACASI survey modality, with pre-recorded voice prompts in Kiswahili, running from April to July of 2022. This period coincided with data collection for the 2022 DHS, which ran from February 2022 to July 2022. As in 2017–18, both surveys solicited information from respondents for the calculation of select RBM-MERG ITN coverage indicators. However, the 2022 MPS used a streamlined survey focusing specifically on the minimum number of data elements needed to calculate bed net coverage indicators (*e.g.*, "How many people usually live in your house?"; "How many mosquito nets does your household own?") and to perform survey raking (*i.e.*, questions about durable goods ownership). Knowing that the vast majority of nets in Tanzania are insecticide-treated [20, 21], the 2022 MPS did not require respondents to distinguish between treated and untreated bed nets when reporting counts, as was the case in the 2018 MPS. Additionally, the 2022 MPS omitted drill-down style questions that solicited information on counts of sub-groups within the household (*i.e.*, children under five, adults aged 18 and over), details on SIM cards and mobile phone ownership within the household, and indoor residual spraying. These questions were included in the 2018 MPS but were excluded in the streamlined 2022 version to mitigate the occurrence of respondent drop-offs.

Unlike the 2018 MPS, the 2022 MPS utilized a “known number” or list-based approach, sampling respondents from an opt-in pool. Vodacom, a major mobile network operator in Tanzania, provided Viamo, the mobile phone survey operator, with a database of phone numbers of subscribers who had opted into a programme that offered opportunities to participate in phone-based surveys related to health and well-being. The database included information on geographical location, thereby shortening the number of cascade-style questions necessary to obtain a respondent’s region of residence. In utilizing the known number survey approach, the call centre randomly sampled from geographic zone-specific blocks of numbers until targeted regional quotas were met.

The date that a Vodacom subscriber opted into the known number survey programme was recorded and Viamo was able to prioritize more recent blocks of numbers for use in the survey. In the event there was difficulty reaching targeted sample sizes, Viamo was able to sample from older participant rolls. Efforts were made to use numbers from those respondents who had opted into the known number survey programme within the 12 months preceding the survey. Vodacom’s market share comprises approximately 30% of all telecom subscriptions across Tanzania [41].

Another difference in the way the two mobile phone surveys were conducted was related to how regional sample sizes were met. The 2018 MPS did not require that all questions necessary for calculating the RBM-MERG ITN coverage indicators be answered for the survey to count towards regional sample size quotas. The 2022 MPS, however, did require that these questions be answered in order for the survey to count toward the sample size quotas.

### Sample size

The sample size targets for both the 2018 and 2022 MPS were based on a quota-based sampling strategy, in which mobile phone calls were placed until a target number of completed interviews per region was achieved. This approach allowed us to absorb non-response into the fieldwork process rather than into the analytic sample size calculation. A target of 250 completed interviews per region was selected to ensure that the precision of ITN coverage estimates from the MPS would be comparable to that of the 2017 and 2022 DHS (e.g., such that 95% confidence intervals around point estimates would not overlap if the difference in proportions was greater than twice the absolute precision of the DHS estimate). Assuming a baseline of 80% ownership of at least one ITN at the household level, a sample of 250 respondents per region was expected to provide approximately 88% power to detect a 10-percentage point difference under the assumption of simple random sampling. Because the MPS did not involve geographic or household clustering, a design effect adjustment based on intra-cluster correlation was not applied.

### Statistical methods

Data were cleaned and analysed using R 4.0.1 [42]. In the event multiple contact attempts were made during the MPS survey, the following guidance was adapted from the American Association of Public Opinion Research (AAPOR) to de-duplicate calls: 1) if one of separately placed calls to the same number resulted in human contact, it took precedence, 2) if one of separately placed calls to the same number resulted in more responses to survey questions, it took precedence, and 3) if separately placed calls to the same number provided the same information to each survey question, the earliest call took precedence. The AAPOR has established standard definitions for use in describing contact, response, cooperation, and refusal rates from telephone surveys [43]. Descriptive statistics were calculated for these AAPOR definitions and other survey attributes.

To be dispositioned as a completed survey, respondents needed to answer all questions used to calculate the selected RBM-MERG ITN coverage indicators as well as those questions used for survey raking. Respondents of minimally completed surveys only needed to answer those questions used to calculate the coverage indicators. Respondents who finished the survey but skipped any of the questions used to calculate the coverage indicators had their call dispositioned as ‘other’. Respondents with calls dispositioned as break off surveys did not finish the survey and answered some combination of the questions used to calculate the coverage indicators but left at least one blank. Respondents who chose not to respond to the first survey question had their call dispositioned as a refusal—these respondents stayed on the call long enough for the MPS call system to pose the first question of the survey before they ended the call. Calls during which no response was recorded for any question were dispositioned as non-contacts—these respondents did not stay on the call long enough for the system to pose the first question.

To ensure valid estimation of ITN indicators, records with missing or logically inconsistent responses on key variables, including household size, number of ITNs owned, and ownership of durable goods were excluded. These exclusions were limited to item-level issues and did not reflect broader unit-level non-response. Imputation techniques were not used, as the missingness was not assumed to be missing at random and the inconsistencies could not be resolved through deterministic correction. The number of records excluded due to data quality concerns is reported in the Results section. These exclusions were necessary to preserve the integrity of indicator definitions and avoid distortions in the regional estimates used for comparison with DHS and MIS values.

RBM-MERG ITN indicators were calculated for both the 2018 and 2022 MPS and the DHS household surveys at the regional level to describe and compare estimates of ITN coverage and access. ITN use was excluded from analysis due to the relative paucity of information collected on net users primarily as a result of illogical responses, drop-offs, and skipped questions. The three remaining coverage indicators were calculated for any type of bed net present in the household (treated and untreated alike).

Survey raking via iterative post-stratification was used to match the marginal distributions of the MPS samples with the known population margins from the DHS surveys [44]. These margins included ownership of radio, TV, bicycle, and car/truck; household size; and region. This process aimed to improve the comparability of the MPS samples with those of the DHS.

For each assessed indicator, two separate analyses were conducted to compare regional-level MPS estimates to their contemporaneous DHS estimates. First, raked and unraked regional survey point pairs (MPS vs DHS) were plotted for each assessed indicator, with regression lines fitted to visually demonstrate the linear relationship between the survey estimates. This approach provided a measure of relative bias, with a single slope value representing the proportional agreement or bias across all survey point pairs for each indicator. Both MPS raked and inverse variance weighted as well as MPS unraked and unweighted regression lines are depicted for each indicator. Accompanying this graphic are a series of simple linear regression analyses where the dependent variable is the DHS regional estimate, and the independent variable is the corresponding MPS regional estimate.

Second, Bland–Altman analyses were performed to evaluate the overall degree of bias between the two survey types. In these plots, the x-axis represents the mean proportion of each regional estimate pair (MPS vs DHS), and the y-axis represents the difference between these proportions. This method provided a measure of absolute bias by directly calculating the differences between survey estimates, ensuring that the assessment is not influenced by proportional effects or regression to the mean. For these reasons, the Bland–Altman analyses were prioritized as the primary method for assessing bias in this work.

### School net programme

To help sustain ITN coverage levels in Tanzania, the School Net Programme (SNP) was developed in 2013 as a ‘keep-up’ distribution strategy. This programme distributes ITNs annually through the primary school system in certain regions of Tanzania. Between August and November 2017, the fifth round of the SNP (SNP-5) distributed just over three million nets to school-going children across 14 regions [45]. The 2017 MIS was conducted before SNP-5 began in most regions of Tanzania, but six regions (Geita, Katavi, Kigoma, Morogoro, Shinyanga, and Simiyu) experienced school-based ITN distribution in a concurrent manner with or after the MIS was completed. In a clearly indicated fashion, certain tables and figures entirely exclude these six regions to account for instances where SNP-5 distribution occurred during or after the MIS, but before the RDD survey began.

### RBM-MERG indicators

The standard RBM-MERG indicators used for comparison across surveys are:

1. Proportion of households with at least one bed net of any type
  - Numerator: Number of households surveyed with at least one bed net of any type
  - Denominator: Total number of households surveyed
2. Proportion of households with at least one bed net of any type for every two people
  - Numerator: Number of households with at least one bed net of any type for every two people (people to net ratio of ≤ 2)
  - Denominator: Total number of households surveyed
3. Proportion of population with access to a bed net of any type in their household
  - Numerator: Total number of individuals who could sleep under a bed net of any type if each net in the household were used by two people
  - Denominator: Total number of individuals who spent the previous night in surveyed households

### AAPOR standard definitions

AAPOR standard definitions are used to describe the contact (CON3), response (RR5 and RR6), cooperation (COOP1 and COOP2), and refusal/break-off (REF3) rates for the RDD survey [43]. These measures are defined below where I = complete interview, M = minimally complete interview, R = refusal and break off, NC = non-contact, and O = other:

$$\text{CON}3=\frac{\left(I+M\right)+R+O}{\left(I+M\right)+R+O+NC}$$

$$\text{RR}5= \frac{I}{\left(I+M\right)+\left(R+NC+O\right)}$$

$$\text{RR}6= \frac{I+M}{\left(I+M\right)+\left(R+NC+O\right)}$$

$$\text{COOP}1=\frac{I}{\left(I+M\right)+R+O}$$

$$\text{COOP}2=\frac{(I+M)}{\left(I+M\right)+R+O}$$

$$\text{REF}3=\frac{R}{\left(I+M\right)+\left(R+NC+O\right)}$$

### Ethical clearance

Ethical clearance for the 2017 MPS was obtained from the National Institute for Medical Research Ethics committee (NIMR/HQ/R.8a/Vol. IX /2640), the Ifakara Health Institute Institutional Review Board (IHI/IRB/No: 23–2017), and the Tulane University Institutional Review Board (2017–613). Ethical clearance for the 2022 MPS was received from the Tanzania National Institute for Medical Research (Ref. NIMR/HQ/R.8a/Vol. IX/3473). Participant consent was obtained from an affirmative response to a pre-recorded message that read out a short consent script at the beginning of the mobile phone survey as well as confirmation that the respondent was over the age of 18 years. Phone numbers were included in the initial datasets but were replaced by randomly generated unique identifiers such that dataset records were not identifiable.

## Results

The 2017 Tanzania MIS and 2022 Tanzania DHS interviewed respondents from 9,330 and 15,705 households, respectively (full details are included in the publicly available reports at https://dhsprogram.com). Both the MIS and DHS were designed to provide regionally representative results for all 31 regions of Tanzania. The 2018 MPS was only able to provide ITN coverage estimates for 24 regions (Pwani, Songwe, and Tanga regions were inadvertently excluded from the RDD process). Also, due to the small number of completed interviews, all regions of Pemba and Unguja islands were combined to form a single region for all of Zanzibar. With the exclusion of SNP-5 affected regions, data presentation for the 2018 MPS focuses on a total of 18 regions. Similar to the approach taken for the 2018 MPS, Pemba and Unguja islands were combined as one region for the 2022 MPS; otherwise, all regions of Mainland Tanzania were included in the analysis of the 2022 MPS.

The 2018 MPS placed 34,821 calls, 95.2% of which were unique (Table 1). Just over two-thirds (68.8%) of all placed calls resulted in successful contact (CON3) with a respondent, but the overall response rate for complete interviews (RR5) was 6.5%. The majority (54.3%) of placed calls were dispositioned as refusals or break offs (REF3) meaning a respondent answered the call but ended it before enough questions were answered to constitute even a minimally complete survey. Note that minimally complete survey designations only required that participants answer the questions necessary for calculation of the bed net coverage indicators of interest, but not those used for survey raking. For the 2018 MPS, any call with usable information was taken into consideration for the calculation of each indicator regardless of call disposition status. This may have resulted in differing sample sizes for the assessed indicators even within the same region.

**Table 1:** Mobile phone survey call characteristics and AAPOR call disposition

	2018 MPS	2022 MPS
Calls		
Total calls placed	34,821	120,262
Total unique calls placed	33,137 (95.2%)	48,738 (40.5%)
Call disposition		
(NC) Non-contacts	10,338 (31.2%)	22,644 (44.7%)
(R) Refusals and break offs	18,003 (54.3%)	13,155 (27.0%)
(M) Minimally complete	2,602 (7.9%)	583 (1.2%)
(I) Complete interviews	2,165 (6.5%)	6,787 (13.9%)
(O) Other	29 (0.1%)	1,648 (3.4%)
Ineligible respondents*	NA	3,921 (8.0%)
AAPOR call disposition		
Contact rate [CON3]: (I + M) + R + O/(I + M) + R + NC + O	68.8%	49.5%
Response rate [RR5]: I/((I + M) + (R + NC + O))	6.5%	15.1%
Response rate [RR6]: (I + M)/((I + M) + (R + NC + O))	14.4%	16.4%
Cooperation rate [COOP1]: I/(I + M) + R + O)	9.5%	30.6%
Cooperation rate [COOP2]: (I + M)/((I + M) + R + O))	20.9%	33.2%
Refusal rate [REF3]: R/((I + M) + (R + NC + O))	54.3%	29.3%
Average survey length		
Complete interviews	9 min 10 s	4 min 01 s

^*^Not an official AAPOR designation – ineligible respondents were under the age of 18 years or were excluded from participation because their call exceeded the regional quota. In 2018, the mobile phone survey operator did not include/record ineligible respondents in the data set provided to the authors

*AAPOR* American Association of Public Opinion Research; *MPS* mobile phone survey; *min* minutes; *sec* seconds

The 2022 MPS placed a total of 120,263 calls to 48,738 unique phone numbers (Table 1). Of the total calls placed, 44.7% (n = 22,644) of non-contacts were recorded alongside 3921 calls determined to be made to ineligible respondents (i.e., ineligible respondents were under the age of 18 years or were excluded from participation because their call exceeded the regional quota). In total, 6787 interviews were designated as complete, meaning all bed net indicators of interest for these respondents were calculable and for which survey raking was possible. Because the known number method was expected to have fewer non-contact calls by virtue of the opt-in nature of the programme, the cooperation rate (COOP1) can be assessed to get an alternative indication of successful interviews as non-contacts are removed from the denominator. This metric was calculated at 30.6% for complete interviews. The average survey length for completed calls was approximately five minutes shorter in the 2022 MPS, with an average length of 4 min and 1 s, compared to the 2018 MPS, which had an average length of 9 min and 10 s.

Table 2 shows the RBM MERG ITN ownership and access indicators by region for mainland Tanzania and Zanzibar for all surveys. It also includes the number of usable calls by region. Excluding SNP-5 affected regions, nearly three quarters (13 of 18 regions; 72.2%) of regional estimates derived from the 2018 MPS underestimated the indicator for household ownership of at least one bed net of any type when compared to the reference standard MIS. Six of 18 regions (33.3%) had 2017 estimates for this indicator that were less than or equal to two percentage points from the corresponding MIS estimates. The 2022 survey estimates for this indicator showed that 21 of 27 regional estimates (77.8%) were underestimated compared to the 2022 DHS with an average percentage point difference of − 12.2%. The 2022 regional MPS estimates for Geita, Katavi, Lindi, and Tanga were all approximately 20 percentage points lower than the corresponding DHS estimates for this same indicator.

**Table 2:** Survey raked estimates for RBM-MERG indicators for nets of any type, by region and survey

Region	Households with at least one bed net of any type						Households with at least one bed net of any type for every two people						Population with access to a bed net of any type in their household					
	2017–18			2022			2017–18			2022			2017–18			2022		
	N	MPS	MIS	N	MPS	DHS	N	MPS	MIS	N	MPS	DHS	N	MPS	MIS	N	MPS	DHS
Arusha	79	0.63	0.74	289	0.61	0.49	76	0.49	0.54	289	0.37	0.23	76	0.45	0.68	289	0.47	0.34
Dar es Salaam	437	0.83	0.91	255	0.71	0.77	422	0.66	0.69	255	0.50	0.51	422	0.67	0.82	255	0.61	0.69
Dodoma	93	0.77	0.79	275	0.68	0.87	89	0.57	0.50	275	0.41	0.60	89	0.60	0.65	275	0.54	0.76
Geita*	64	0.70	0.78	214	0.64	0.84	59	0.47	0.28	214	0.36	0.32	59	0.57	0.53	214	0.43	0.60
Iringa	43	0.67	0.68	246	0.70	0.78	41	0.49	0.39	246	0.55	0.57	41	0.60	0.56	246	0.57	0.71
Kagera	71	0.87	0.86	272	0.75	0.79	67	0.67	0.55	272	0.54	0.44	67	0.73	0.72	272	0.59	0.66
Katavi*	27	0.81	0.72	247	0.71	0.93	26	0.73	0.34	247	0.51	0.58	26	0.62	0.49	247	0.58	0.80
Kigoma*	57	0.75	0.77	261	0.67	0.73	46	0.57	0.35	261	0.43	0.36	46	0.56	0.59	261	0.47	0.58
Kilimanjaro	89	0.79	0.89	281	0.63	0.60	86	0.63	0.70	281	0.44	0.39	86	0.62	0.81	281	0.47	0.51
Lindi	65	0.85	0.84	231	0.63	0.83	62	0.69	0.63	231	0.47	0.63	62	0.76	0.74	231	0.50	0.75
Manyara	45	0.73	0.77	236	0.64	0.55	38	0.55	0.36	236	0.37	0.24	38	0.61	0.58	236	0.49	0.38
Mara	88	0.76	0.92	269	0.69	0.87	77	0.52	0.51	269	0.39	0.43	77	0.65	0.74	269	0.54	0.65
Mbeya	118	0.77	0.78	299	0.68	0.80	109	0.59	0.53	299	0.48	0.56	109	0.65	0.65	299	0.54	0.70
Morogoro*	184	0.78	0.91	237	0.65	0.84	173	0.62	0.62	237	0.46	0.49	173	0.58	0.78	237	0.55	0.66
Mtwara	76	0.76	0.84	262	0.71	0.78	70	0.64	0.64	262	0.48	0.55	70	0.68	0.80	262	0.54	0.71
Mwanza	141	0.79	0.88	267	0.73	0.89	137	0.59	0.44	267	0.50	0.49	137	0.67	0.70	267	0.58	0.71
Njombe	33	0.64	0.58	280	0.64	0.56	27	0.44	0.43	280	0.40	0.37	27	0.45	0.52	280	0.53	0.48
Pwani	–	–	0.94	237	0.81	0.84	–	-	0.68	237	0.54	0.59	–	–	0.82	237	0.64	0.72
Rukwa	37	0.68	0.71	248	0.65	0.76	37	0.49	0.40	248	0.40	0.40	37	0.44	0.51	248	0.48	0.59
Ruvuma	68	0.76	0.83	252	0.59	0.76	64	0.61	0.60	252	0.38	0.47	64	0.61	0.74	252	0.42	0.64
Shinyanga*	82	0.74	0.76	244	0.74	0.64	77	0.56	0.38	244	0.52	0.27	77	0.57	0.54	244	0.61	0.47
Simiyu*	50	0.78	0.82	212	0.67	0.54	48	0.50	0.33	212	0.39	0.17	48	0.48	0.54	212	0.50	0.35
Singida	49	0.59	0.69	287	0.64	0.73	43	0.47	0.28	287	0.44	0.39	43	0.44	0.43	287	0.51	0.56
Songwe	–	–	0.78	231	0.62	0.64	–	–	0.49	231	0.34	0.35	–	–	0.67	231	0.43	0.51
Tabora	120	0.75	0.73	249	0.71	0.80	106	0.56	0.32	249	0.51	0.29	106	0.55	0.51	249	0.58	0.52
Tanga	–	–	0.90	236	0.67	0.86	–	–	0.52	236	0.45	0.51	–	–	0.74	236	0.51	0.68
Zanzibar	49	0.63	0.81	170	0.68	0.79	46	0.50	0.44	170	0.39	0.52	46	0.49	0.64	170	0.51	0.68

The 2017 MPS erroneously excluded Tanga, Pwani, and Songwe regions. Regions with an asterisk were those which experienced ITN distribution from the SNP-5 during field work for the 2017 MIS but before the 2018 MPS began. MPS estimates in this table are raked

*MPS* mobile phone survey; *MIS* Malaria Indicator Survey; *DHS* Demographic and Health Survey; *SNP* School Net Programme

Household ownership of at least one bed net of any type for every two people was generally overestimated by the 2018 MPS (15 of 18 regions; 83.3%) although four regional estimates (22.2%) were only about one percentage point away from the corresponding 2017 MIS estimates. Excluding SNP-5 regions, the largest difference was noted for Tabora which had a regional estimate that was 23 percentage points higher than the MIS. The 2022 MPS had nearly equal numbers of under- and overestimated regional values for this indicator with almost half (12 of 27 regions; 44.4%) being less than or equal to five percentage points from their corresponding 2022 DHS estimates.

A little over half of the regional estimates (11 of 18 regions; 61.1%) for population with access to a bed net of any type in their household were underestimated by the 2018 MPS when compared to the 2017 MIS, and all seven regional overestimations were within four percentage points of the corresponding MIS values. The 2022 MPS tended to overestimate this indicator (21 of 27 regions; 77.8%) by an average of 13 percentage points relative to the 2022 DHS. The largest differences were noted for Lindi, Katavi, Ruvuma, and Dodoma regions which all had regional estimates 22 to 25 percentage points lower than the corresponding 2022 DHS regional estimates.

**Fig. 1:**
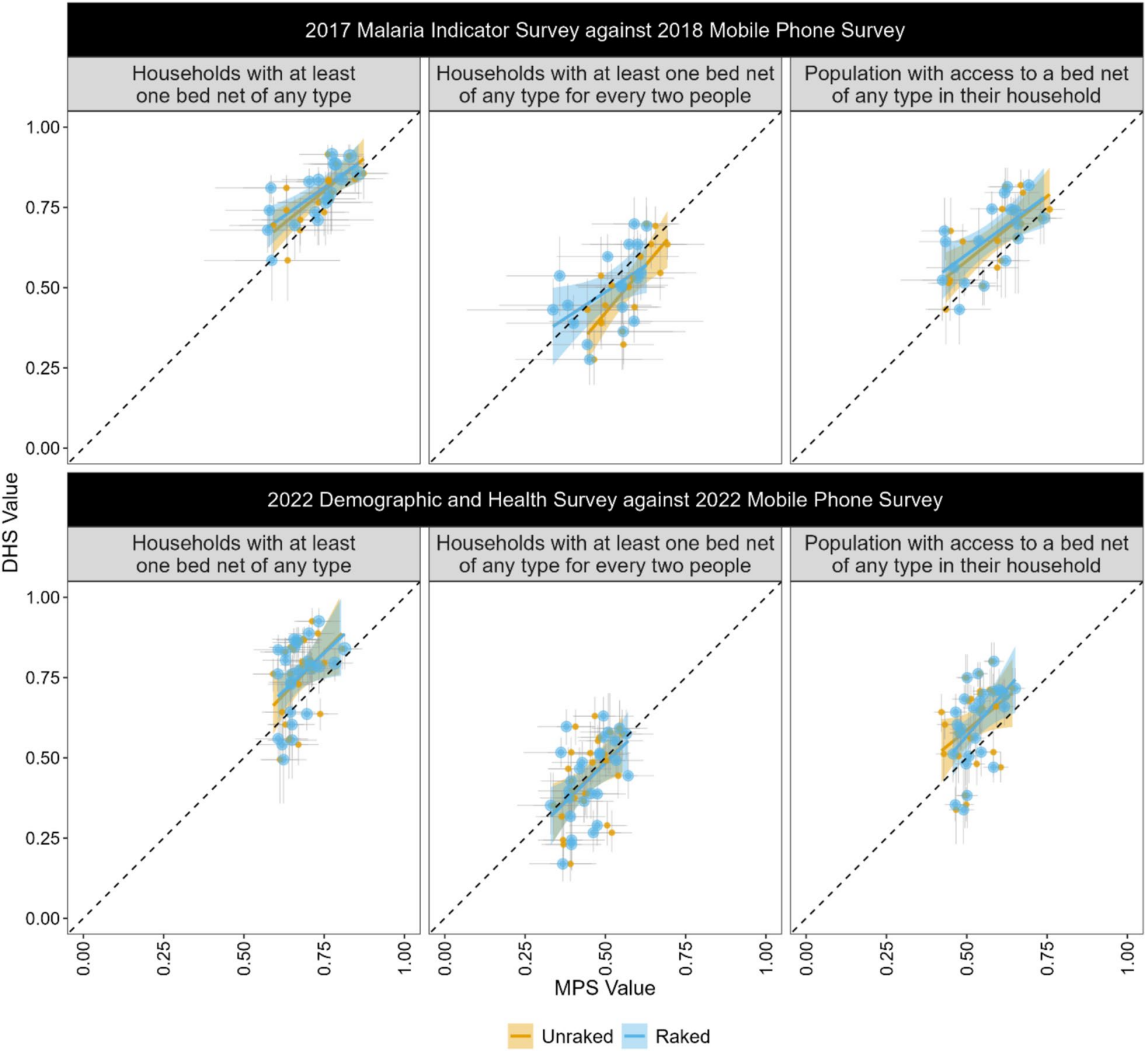
Simple linear regression plots of MPS values against MIS values, by survey pair and indicator. Blue regression lines are based on survey raked and inverse variance weighted MPS estimates; orange regression lines are based on unraked and unweighted MPS estimates. SNP-5 affected regions are excluded from the presentation of the 2017–18 survey data. *DHS* Demographic and Health Survey; *MPS* Mobile phone survey; *SNP* School Net Programme

Raked and unraked regional survey point pairs are plotted in Fig. 1**.** Apart from the 2018 MPS indicator for the proportion of households with at least one bed net of any type for every two people, regression lines for the raked and unraked survey estimates appear to be relatively similar. Most regression lines appear parallel and are relatively close to the 45˚ line of equality upon which a point would fall if the MPS and DHS values were the same. Regression lines for the proportion of households with at least one bed net of any type for every two people in 2022 appear to be superimposed on the line of equality although the survey point pairs demonstrate considerable spread above and below.

Results from the simple linear regression analyses (Table 3) show that raking systematically altered the slopes for most indicators, reflecting adjustments to the MPS sample to align with population margins from the DHS. All three indicators for the 2018 MPS and two of the three indicators for the 2022 MPS showed reduced slopes after weighting. The raked slopes for households with at least one bed net of any type and households with at least one bed net of any type for every two people were underestimated by both the 2018 and 2022 MPS estimates, although the degree of underestimation was smaller in 2022. In contrast, the raked slope for population with access to a net of any type in their household was underestimated by 28% in 2018 and overestimated by 16% in 2022. Adjusted R-squared values were low for each indicator, with none exceeding 0.5.

**Table 3:** Simple linear regression of raked and unraked regional MPS estimates on regional DHS estimates, by indicator and survey year

	2018 MPS (N = 18)				2022 MPS (N = 27)			
	Unraked MPS estimate [95% CI]	Adj R^2^	Raked MPS estimate [95% CI]	Adj R^2^	Unraked MPS estimate [95% CI]	Adj R^2^	Raked MPS estimate [95% CI]	Adj R^2^
Households with at least one bed net of any type								
(Intercept)	0.19 [− 0.11, 0.49]	0.50	0.27 [0.00, 0.55]	0.50	0.04 [− 0.58, 0.66]	0.16	0.16 [− 0.37, 0.69]	0.16
MPS survey value	0.81 [0.41, 1.22]		0.72 [0.35, 1.08]		1.05 [0.13, 1.96]		0.88 [0.11, 1.66]	
Households with at least one bed net of any type for every two people								
(Intercept)	− 0.17 [− 0.51, 0.16]	0.31	0.16 [− 0.14, 0.47]	0.31	0.00 [− 0.33, 0.33]	0.23	0.00 [− 0.30, 0.30]	0.23
MPS survey value	1.19 [0.60, 1.78]		0.65 [0.06, 1.23]		0.97 [0.23, 1.71]		0.96 [0.30, 1.63]	
Population with access to a bed net of any type in their household								
(Intercept)	0.18 [− 0.08, 0.44]	0.43	0.24 [− 0.03, 0.52]	0.43	0.18 [− 0.26, 0.61]	0.18	− 0.01 [− 0.44, 0.41]	0.18
MPS survey value	0.80 [0.38, 1.23]		0.72 [0.26, 1.19]		0.82 [− 0.01, 1.64]		1.16 [0.37, 1.96]	

SNP-5 affected regions are excluded from the 2018 survey data

*MPS* mobile phone survey; *MIS* Malaria Indicator Survey; *DHS* Demographic and Health Survey; *Adj* adjusted; *SNP* School Net Programme

**Fig. 2:**
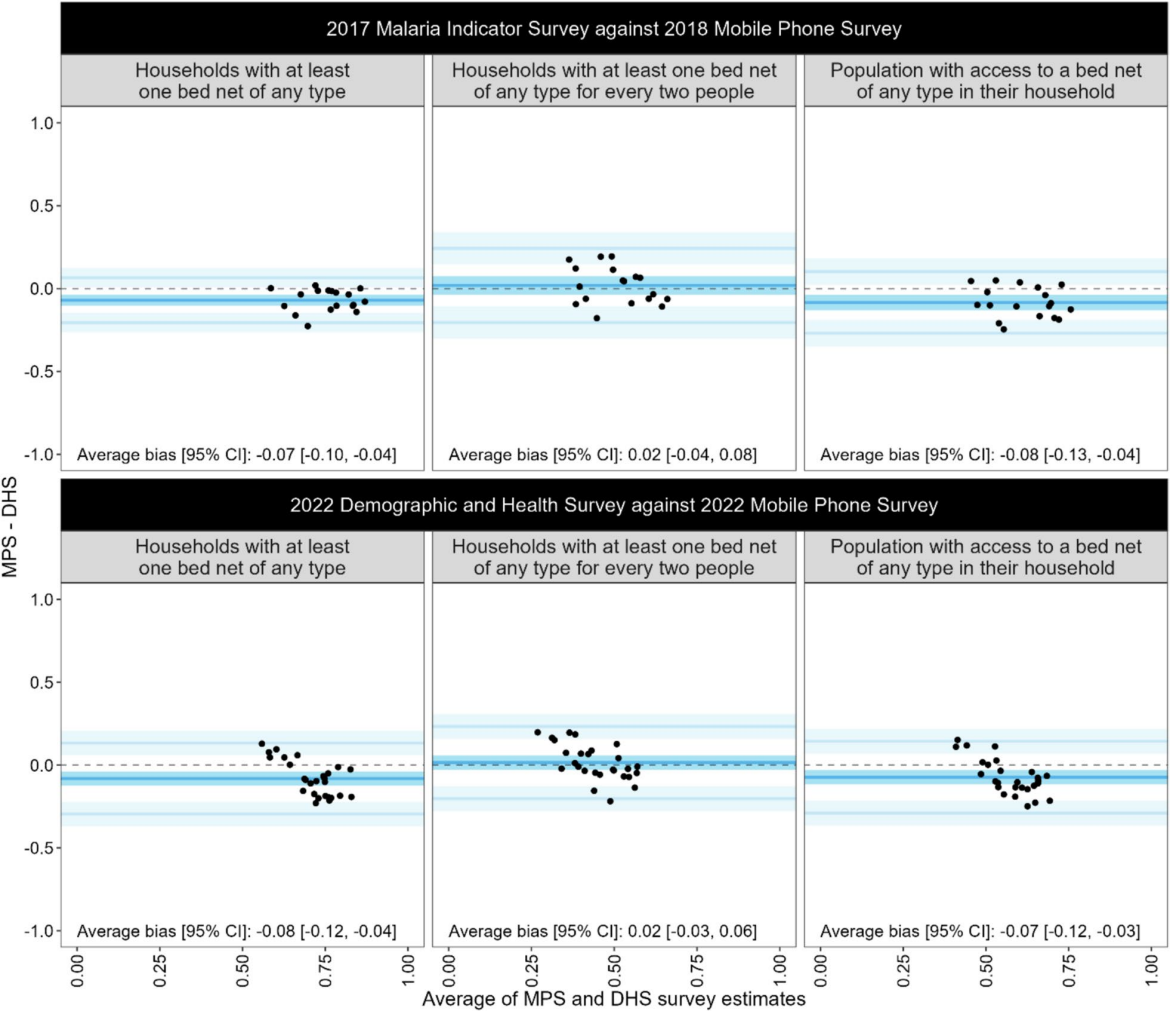
Bland–Altman plots, by survey pair and indicator. Plots make use of survey raked MPS estimates. The black dotted line (y = 0) represents zero bias between the two measures. The dark blue line, with confidence intervals in dark blue, depicts the mean bias between the two measures. Light blue areas represent the 95% confidence intervals for the limits of agreement (± 1.96 times the standard deviation of the differences). SNP-5 affected regions are excluded from the presentation of the 2017–18 survey data. *MPS* mobile phone survey; *DHS* Demographic and Health Survey; *CI* confidence interval; *SNP* School Net Programme

Bland–Altman analyses measuring absolute bias for each assessed indicator are presented in Fig. 2. For both the 2018 and 2022 surveys, the measures of bias were negative for households with at least one bed net of any type and population with access to a bed net of any type in their household. Specifically, the mean measures of bias for the 2018 survey were − 0.07 (95% CI [− 0.10, − 0.04]) and − 0.08 (95% CI [− 0.13, − 0.04]), respectively, while for the 2022 survey, they were − 0.08 (95% CI [− 0.12, − 0.04]) and − 0.07 (95% CI [− 0.12, − 0.03]). These results indicate that, on average, MPS estimates for these indicators were consistently lower than the corresponding household survey estimates. In contrast, the indicator for households with at least one bed net of any type for every two people had mean measures of bias that were 0.02 (95% CI [− 0.04, 0.08]) for 2018 and 0.02 (95% CI [− 0.03, 0.06]) for 2022, indicating that the MPS provided unbiased estimates for this indicator, following raking, relative to the DHS estimates.

Among the completed surveys from the 2022 MPS, 1411 records had illogical responses related to net use (*e.g.*, more net users reported than total household members). These records were removed for the calculation of all indicators given the inability to verify responses related to the calculation of net ownership and access. A sensitivity analysis was conducted which included these records and showed that the mean bias increased from − 0.08 to − 0.10 for households with at least one bed net of any type and from − 0.07 to − 0.12 for population with access to a bed net of any type in their household. The mean bias for households with at least one bed net of any type for every two people decreased slightly from 0.01 to 0.00.

## Discussion

The primary objective of this study was to assess the external validity of two TACASI MPS conducted in 2018 and 2022, each utilizing a distinct sampling methodology, by comparing their estimates to those from contemporaneous, nationally representative household surveys (the 2017 MIS and the 2022 DHS, respectively). The external validity of the MPS-derived estimates varied by ITN indicator and geographic level of interpretation, with both surveys consistently underestimating household access to a bed net of any type. While this underestimation highlights important limitations, particularly for NMCPs seeking accurate benchmarks, it also underscores the potential for MPS to serve as an early, conservative signal of declining coverage in some contexts. When interpreted with an understanding of their biases and the conditions that support accuracy (e.g., high mobile penetration and widespread ITN access), MPS may still help flag emerging gaps in coverage or support planning where other data are delayed or unavailable.

Despite methodological refinements in the 2022 MPS, including a shift from RDD to a known-number approach and a shorter, more targeted questionnaire, nearly identical average measures of bias were observed compared to the 2017 MPS. These findings suggest that improvements in design alone may not be sufficient to overcome structural limitations of mobile phone-based sampling. In particular, mobile phone ownership in Tanzania remains more common among wealthier, urban, and more educated households, and disproportionately higher among men than women [46]. This skews the respondent pool and can limit representativeness, particularly in settings where coverage gaps are patterned along these dimensions. Moreover, studies have shown that male and female co-heads of households sometimes provide divergent responses to basic questions about household composition and assets [47–51]. If male respondents, who are more likely to own phones, lack accurate knowledge of household size or ITN ownership, this could introduce additional response error into MPS-derived estimates. As mobile access continues to expand across Tanzania, however, the accuracy and reliability of MPS estimates may improve, reducing non-coverage bias and strengthening their value as a monitoring tool.

Concerns about sampling bias, specifically related to the exclusion of rural or lower-SES households, may not explain the observed downward bias. Prior analysis using DHS data found that phone-owning households in Tanzania tend to have higher ITN access than their non-owning counterparts, particularly in settings with declining national coverage [46]. This suggests that the MPS sample frame, if anything, is skewed toward higher-coverage households, which should upwardly bias results. The fact that underestimation persists suggests alternative sources of measurement error, such as respondent fatigue, challenges navigating IVR menus, or misreporting of numeric values used to calculate derived indicators. Small inaccuracies in these inputs can produce non-trivial distortions in the resulting access estimates, especially when data are weighted through raking procedures. These findings point to the need for further refinements to survey design, particularly around instrument length and clarity, and may warrant consideration of alternative modes such as computer-assisted telephone interviews (CATI) to reduce cognitive burden and enhance data quality.

Interpretation of MPS-derived ITN coverage estimates is also influenced by how ITN distribution activities are geographically implemented. In Tanzania, for example, while the SNP operates at the regional level and reproductive and child health clinics distribute ITNs to pregnant women and infants [52], the 2020 mass replacement campaign was implemented across 52 districts in 10 regions that had not been covered by the SNP. This distribution occurred in such a way that not all districts within implicated regions received ITNs (as was the case for Kilimanjaro, Manyara, and Njombe), resulting in a sub-regional distribution strategy that may have introduced ambiguity into regional-level MPS estimates. Because MPS respondents could have resided in either targeted or untargeted districts within the affected regions, the resulting MPS estimates likely obscured regional variation in ITN coverage. This underscores the importance of considering campaign design when interpreting survey-based regional coverage estimates.

Beyond issues of representativeness, response rates remain a persistent operational challenge for MPS. The 2022 survey employed a known-number approach and a streamlined questionnaire, resulting in a higher complete interview response rate (15.7 percent) compared to the 2018 MPS (6.5 percent). This improvement was likely due in part to the redesigned instrument, which was approximately five minutes shorter and limited to only the data required to calculate RBM-MERG indicators and conduct survey raking.

While the 2018 MPS response rate of 6.5 percent for complete interviews was not entirely unanticipated, it was quite low when compared to the 21 percent response rate reported by the authors of a nationally representative RDD IVR MPS conducted in Ghana in 2017 [40]. That study was the only one of its kind to be conducted in an LMIC using an RDD methodology to estimate bed net indicators nationally. However, it only assessed ITN use, comparing MPS estimates to the 2014 Ghana DHS conducted three years prior. Given that ITN coverage indicators can vary significantly over time due to large-scale distributions and subsequent net attrition, it is not reasonable to expect consistency between surveys conducted at such different time points.

A separate RDD study conducted in Côte d’Ivoire in 2013 used a CATI methodology to collect data on HIV/AIDS screening [53]. Indicator estimates from this study, which relied on live interviewers, were compared to similar indicators from the 2011–2012 DHS to assess external validity. The survey auto-dialed 2,700 randomly generated mobile numbers and achieved 1,084 completed interviews in 19 days, with an average interview duration of seven minutes. This study required only 2.5 calls per completed survey, compared to six to seven calls per completed survey presented in the results above. This difference in call efficiency highlights the need to explore alternative engagement strategies, such as live interviewers, to improve response rates and optimize MPS performance in different contexts.

Survey break-off is another key concern, particularly for IVR-based surveys. In the 2018 MPS, 13 percent of consenting respondents exited after answering the first question in the cascade used to determine region of residence. This line of questioning was necessary for both survey weighting and regional indicator estimation but likely contributed to early attrition. Minimizing survey length and restricting questions to essential elements for indicator construction and raking should remain a design priority. Compared to other modalities, IVR surveys generally yield lower response and completion rates and are especially susceptible to fatigue caused by repetitive structures or unclear automated voice recordings [32, 54, 55].

One additional challenge in MPS is the potential for satisficing [56–58], a response behavior in which participants provide acceptable but not optimal answers. Although the extent to which satisficing varies by interview mode remains unclear [59–62], respondents are more likely to be engaged and less distracted during in-person interviews. In the current study, histograms of reported household size and ITN counts revealed heaping at values such as 10 and 20, suggesting rounding or estimation. These values were outliers compared to DHS household rosters and ITN listings. Cognitive burden, particularly when questions require exact recall of numeric values, may prompt respondents to rely on heuristic shortcuts [63, 64]. Researchers conducting RDD MPS in Bangladesh and Uganda similarly noted that simpler questions with clear response categories showed higher reliability when comparing responses across IVR and CATI modes [65].

Lastly, indicator estimates produced by surveys are subject to total survey error, which includes both sampling variability as well as non-sampling variability (i.e., bias). While this study presents confidence intervals to quantify sampling error, non-sampling error is more difficult to measure. Previous work has shown that MPS can replicate household survey results under certain conditions, but accuracy is not guaranteed. These findings underscore the importance of continued ground-truthing and validation of MPS data to ensure that they provide accurate estimates for policy and planning.

## Limitations

The number of complete surveys in the 2018 MPS was low across many regions. Rukwa, for example, only had 37 complete interviews, far short of the 250 interviews required to detect a 10-percentage point difference with 88% power. Only Dar es Salaam surpassed this threshold, and the average number of complete surveys across the remaining 17 regions was only 74. Future survey work would need to ensure a more explicit understanding on the part of survey operators that a survey designation of ‘complete’ entails participants responding to all required questions irrespective of whether they stay on the call to the final question. Note that respondents in the 2018 MPS were able to skip questions and still technically finish the survey, but if questions required for survey raking or for the calculation of the assessed RBM-MERG ITN indicators were skipped, the survey could not be designated as ‘complete’ for the purposes of analysis. Additionally, it may be possible to boost response rates by offering airtime incentives to MPS participants as found by researchers who conducted RDD IVR studies in Bangladesh and Uganda [65].

Another limitation concerns the timing of the 2018 MPS which followed the MIS by anywhere from one to five months. Although no major ITN distribution campaigns occurred during this period, some decline in household net ownership is expected due to natural attrition (e.g., damage, loss, disposal). As a result, small discrepancies in ITN coverage indicators between the two surveys are likely possible over this temporal gap. However, given the short interval and the relatively slow rate of net attrition over time, the impact on comparative estimates is likely limited.

## Conclusions

The external validity of the RDD MPS was variable but generally underestimated the key coverage indicator of population with access to a bed net of any type in their household. The measures of bias for each assessed indicator were consistent across years in both direction and magnitude, suggesting the potential to apply a correction factor to MPS estimates. However, additional data points, beyond the two MPS presented in this paper, would be required to validate such an approach before advising malaria programme managers. Furthermore, the extent to which programmatic actions based on MPS estimates with uncorrected bias could lead to detrimental or favorable malaria outcomes remains unknown. An analysis of how a difference in ITN coverage—equivalent to the observed bias of 7–8 percentage points—might influence malaria transmission dynamics is warranted. Finally, as certain country programmes increasingly prioritize sub-national tailoring of malaria prevention and control strategies, the need for large-scale MPS may diminish, given the resource-intensive nature of achieving sub-regional-level sample sizes and the challenges of collecting data for lower-level geographic locations.

## Data Availability

The datasets used and/or analysed during the current study are available from the corresponding author on reasonable request.
